# Incidence and clinical impact of vertebral endplate changes after limited lumbar microdiscectomy and implantation of a bone-anchored annular closure device

**DOI:** 10.1186/s12893-020-01011-3

**Published:** 2021-01-06

**Authors:** Jenny C. Kienzler, Sofia Rey, Oliver Wetzel, Hermien Atassi, Sabrina Bäbler, Felice Burn, Javier Fandino

**Affiliations:** 1 Department of Neurosurgery, Kantonsspital Aarau, Tellstrasse, 5001 Aarau, Switzerland; 2 Department of Health Sciences and Technology, ETH Zurich, Zurich, Switzerland; 3 Neuro Research Office, Neurocenter, Kantonsspital Aarau, Aarau, Switzerland

**Keywords:** Disc herniation, Reherniation, Endplate changes, Annular closure device, Polymer mesh, Barricaid®

## Abstract

**Background:**

An annular closure device (ACD) could potentially prevent recurrent herniation by blocking larger annular defects after limited microdiscectomy (LMD). The purpose of this study was to analyze the incidence of endplate changes (EPC) and outcome after LMD with additional implantation of an ACD to prevent reherniation.

**Methods:**

This analysis includes data from a) RCT study-arm of patients undergoing LMD with ACD implantation and b) additional patients undergoing ACD implantation at our institution. Clinical findings (VAS, ODI), radiological outcome (reherniation, implant integrity, volume of EPC) and risk factors for EPC were assessed.

**Results:**

Seventy-two patients (37 men, 47 ± 11.63yo) underwent LMD and ACD implantation between 2013–2016. A total of 71 (99%) patients presented with some degree of EPC during the follow-up period (14.67 ± 4.77 months). In the multivariate regression analysis, localization of the anchor was the only significant predictor of EPC (p = 0.038). The largest EPC measured 4.2 cm^3^. Reherniation was documented in 17 (24%) patients (symptomatic: n = 10; asymptomatic: n = 7). Six (8.3%) patients with symptomatic reherniation underwent rediscectomy. Implant failure was documented in 19 (26.4%) patients including anchor head breakage (n = 1, 1.3%), dislocation of the whole device (n = 5, 6.9%), and mesh dislocation into the spinal canal (n = 13, 18%). Mesh subsidence within the EPC was documented in 15 (20.8%) patients. Seven (9.7%) patients underwent explantation of the entire, or parts of the device.

**Conclusion:**

Clinical improvement after LMD and ACD implantation was proven in our study. High incidence and volume of EPC did not correlate with clinical outcome. The ACD might prevent disc reherniation despite implant failure rates. Mechanical friction of the polymer mesh with the endplate is most likely the cause of EPC after ACD.

## Background

Limited lumbar microsurgical discectomy (LMD) to remove the free disc fragment is the gold standard treatment for patients with refractory disc herniation related radicular pain [22]. However, the failure rate of discectomy is high due to recurrent disc herniation, progression of degenerative disc disease (DDD) and continuing chronic low back pain [16]. The rate of recurrent herniation is 3%-18% in limited discectomy and correlates among other predictive factors with the annular defect area and percentage of removed disc material [24]. A defect size of > 6 mm wide has a reherniation rate of 27% [6]. Aggressive discectomy on the other hand, may lead to accelerated DDD, instability and disc height collapse with chronic low back pain [24]. Symptomatic recurrent herniation management is either conservative or with repeat surgery including discectomy or fusion [18]. The outcome after repeat surgery is often inferior to primary surgery [9]. An annular closure device (ACD) (Barricaid®, Intrinsic Therapeutics, Inc., Woburn, MA) was recently introduced and could potentially prevent recurrent herniation by blocking larger annular defects after LMD, and preserve the nucleus pulposus within the disc space [19]. The results of a European multicenter randomized controlled trial (RCT) with 550 patients have recently been published. It was shown that the frequency of symptomatic reherniation and reoperation was lower in the ACD compared to the control group—with a similar outcome over a 3-year period [12, 23]. Endplate changes (EPC), however, were more prevalent in the ACD group (84% vs. 30%) [23]. Further post hoc analysis investigating the occurrence of EPC identified mechanical stress from the ACD on the endplates as cause of the significant increase of EPC [5]. There was no correlation with outcome (low back pain or ODI) [5, 14]. The aim of the present study is to share our institution's experience with EPC following LMD with additional implantation of an ACD.

## Methods

### Study design

This analysis includes data from (a) European multicenter RCT study-arm of patients undergoing LMD with implantation of the ACD Barricaid® and (b) additional patients undergoing implantation of commercially available ACD at our institution. Lumbar disc herniation patients scheduled for LMD with a posterior disc height of at least 5 mm were offered ACD implantation. Further information on the RCT study protocol including inclusion and exclusion criteria can be found in the main study publication [23]. Informed consent was obtained for all RCT patients. Prior ethics committee approval was obtained for the multicenter randomized study and for the retrospective data analysis (EC numbers 2012/036; 2016/01740). This study is in accordance with the STROBE guidelines. The results from our study have been previously presented at the AANS conference 2019 [11].

### Outcome measures

X-ray, computed tomography (CT) and magnetic resonance imaging (MRI) was performed prior and at the one year follow up. Clinical outcome assessment included pre- and postoperative score assessment of Visual Analogue Scale (VAS) [10], and Oswestry Disability Index (ODI) [7].

### Volumetry of osteolytic endplate changes

**Fig. 1:**
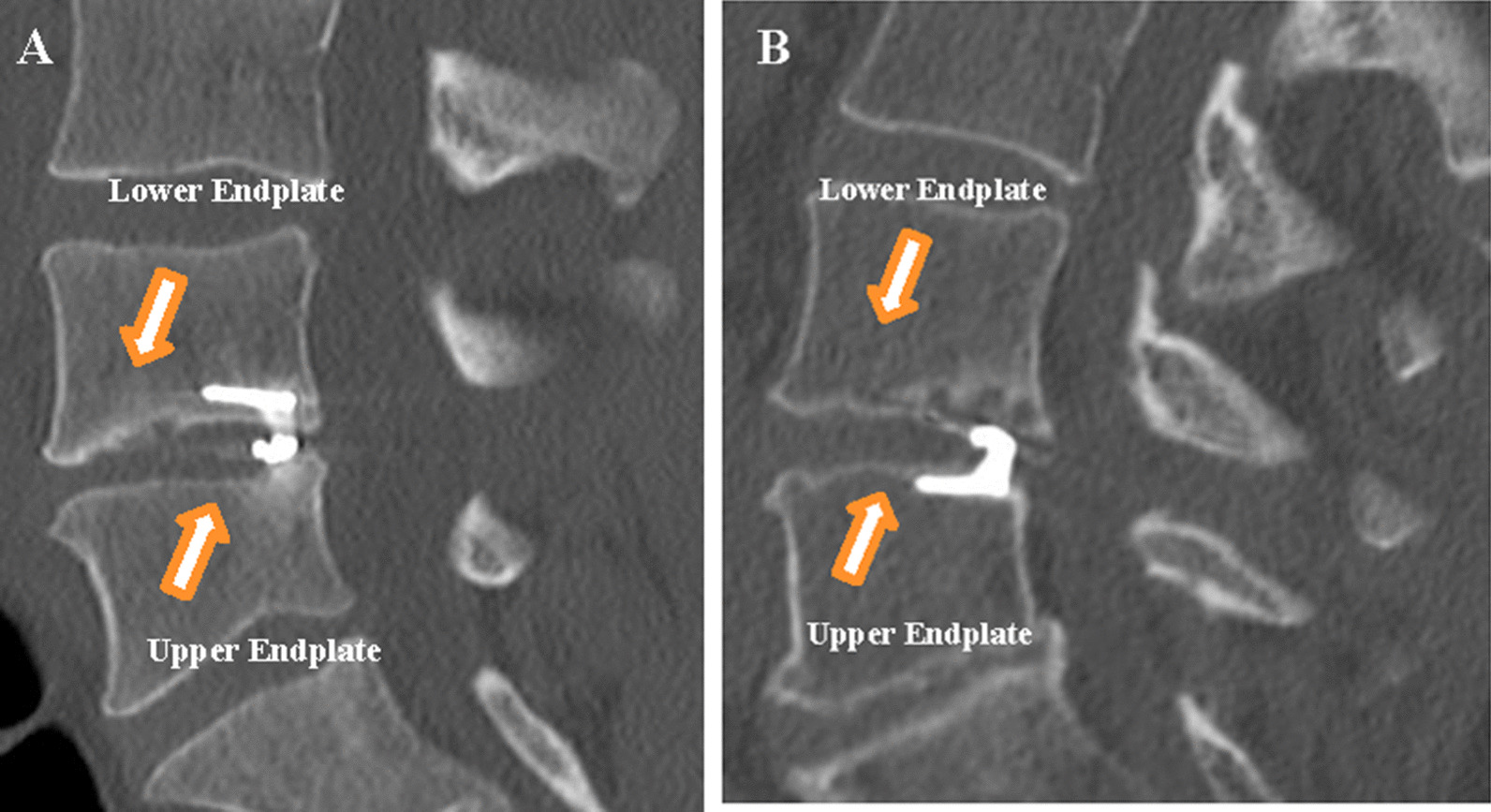
Demonstration of the nomenclature of *upper and lower* endplate changes in relation to the vertebra. **a** ACD anchor implanted in the *inferior* endplate of L4 with EPC in the *upper* endplate of L5. **b** ACD anchor in the *superior* endplate of L5 with EPC in the *lower* EP of L4. *EPC* Endplate changes, *EP* Endplate

**Fig. 2:**
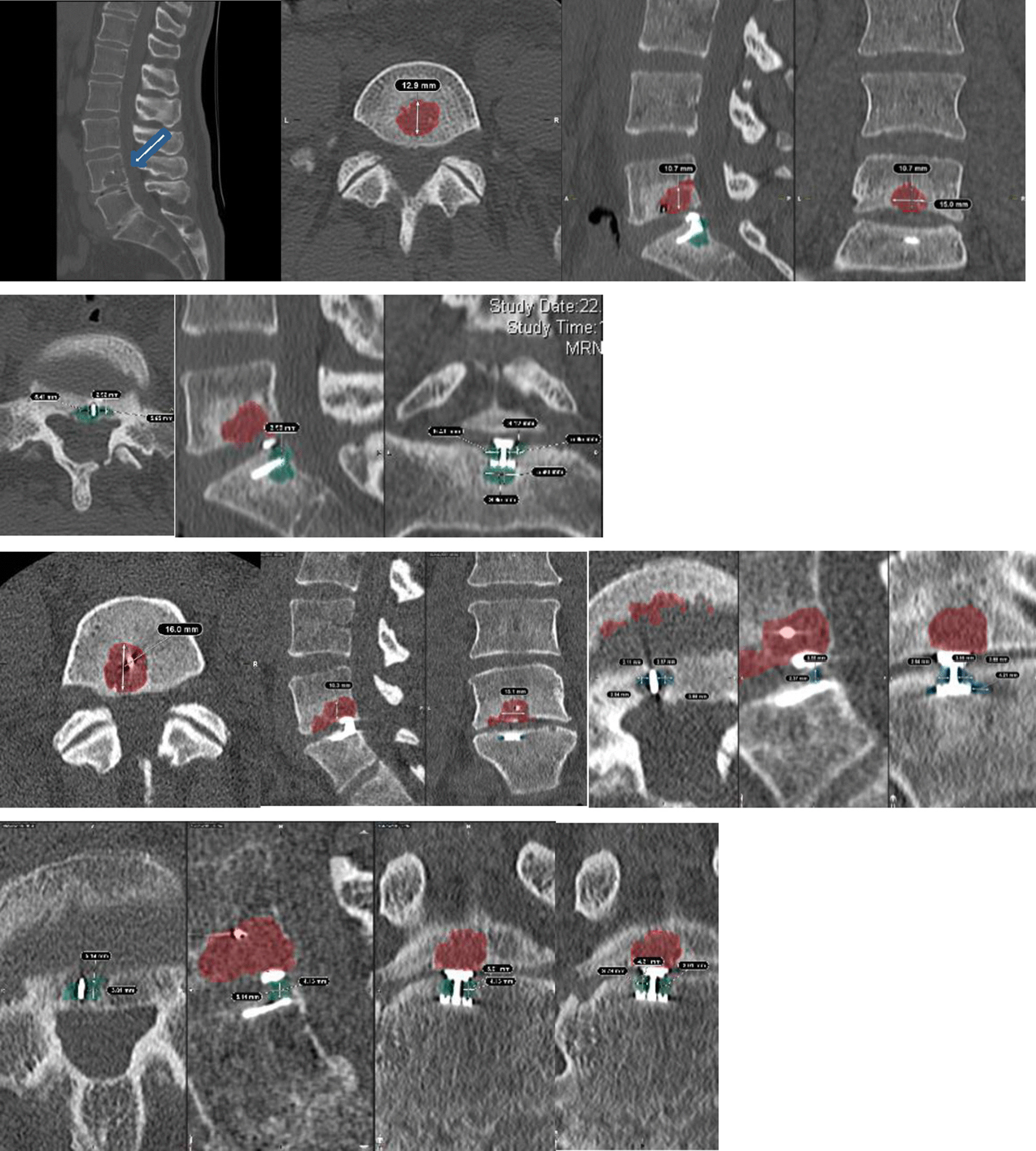
Multiple examples of patients with EPC and the volume measurement technique using Elements software (Brainlab®, Munich, Germany)

Endplate changes (EPC) were classified as any new erosion of subchondral bone, vertebral or cartilaginous endplate. Location of EPC (*lower* or *upper* endplate) and the relation of the titanium device anchor (*superior* or *inferior* endplate) to the vertebra were determined (Fig. 1). Measurements of EPC volume (cm^3^) in the lumbar spine CT were performed using a commercially available software (Elements®, Brainlab, Munich, Germany) (Fig. 2). To increase reliability, all measurements were performed by two neurosurgeons. The interobserver agreement results were accepted for final analysis.

### Endplate and disc degeneration

All EPC were additionally classified according to Modic criteria [17] and DDD was assessed by applying Pfirrmann classification [20]. An independent neuroradiologist conducted all measurements. Disc height was assessed in the pre- and postoperative MRI within 5 mm of the posterior border. In order to rule out measurement errors, 50 randomly selected cases were reevaluated by the first author.

### EPC grading system

We aimed to establish a descriptive grading classification of EPC ranging from 1 to 4 to better assess the extent of EPC and simplify radiological description. The EPC classification includes 2 criteria: localization of device anchor in the vertebra (superior or inferior) and EPC volume (< 1 cm^3^ or > 1 cm^3^).

### Statistical methods

A multivariate regression model was developed in which a linear combination of EPC was regressed on all potential predictive factors to define their significance. Univariate analysis was performed to examine the effect of defined risk factors on the location of EPC (upper vs. lower). The clinical influence of EPC on pain (VAS) and disability scores (ODI) were investigated with a univariate regression analysis of EPC and each outcome parameter. A paired t-test was performed to compare normally distributed continuous data and Wilcoxon rank sum test for non-parametric data.

## Results

### Patient characteristics

A total of 72 patients (37 men) underwent LMD and ACD implantation between January 2013 and October 2016 at a single institution. Twenty-nine patients had surgery and ACD implantation within the randomized controlled trial and 43 were “commercial cases”. The median age at time of surgery was 47 ± 11.63 years. Overall median follow-up was 14.67 ± 4.77 months. Forty-six patients (64%) had smoked at some point in their lives; 33 (46%) of them were still active smokers and 14 (19%) had quit the habit. Forty-seven (67%) patients had a job requiring heavy lifting. The remaining 25 (33%) had an occupation with lighter physical demands and 5 (20%) were already retired.

### Intraoperative Parameters initial surgery

There were no surgical complications. The mean annular defect size was 7.77 ± 1.27 mm in width and 1.35 ± 0.77 cc of disc material was removed on average. The difficulty of surgical procedure with respect to ACD implantation was generally rated as easy or acceptable.

### Endplate changes

**Fig. 3:**
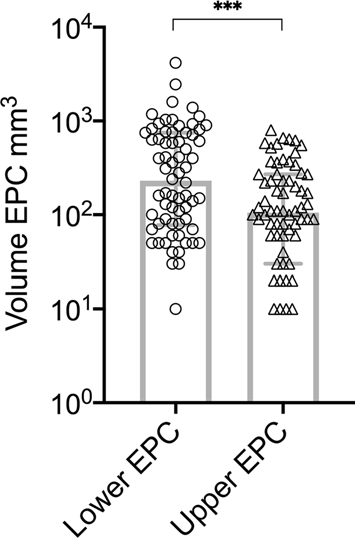
Median EPC volume with interquartile range (25%—75% quartiles) of all patients at the *lower* and *upper* endplate, log(10) scale. p = 0.006

**Fig. 4:**
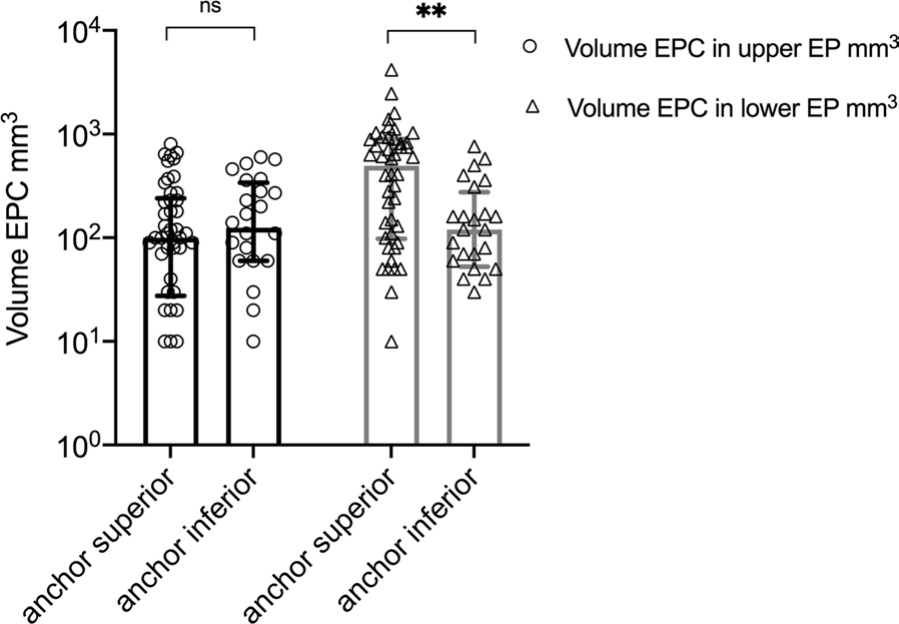
Median *lower* and *upper* EPC volumes with interquartile range (25%—75% quartiles), split out for the two anchor locations *superior* and *inferior*, log(10) scale. p = 0.002

Endplate changes were found in 71 (99%) patients included in this study. These EPC volumes were significantly larger in the *lower* endplate (EP) of the vertebra, with a median of 230 mm^3^, compared with those in the *upper* EP, with a median of 105 mm^3^ (p = 0.006) (Fig. 3). The largest EPC was 4.2 cm^3^. A multivariate regression analysis of risk factors for development of EPC at the *upper* or *lower* endplate only produced one significant predictor: the ACD anchor localization (p = 0.038) (Table 1). Subsequent univariate regression analysis showed that multivariate contribution of anchor localization is based on a significant effect of *lower* EPC after anchor implantation in the *superior* EP (p = 0.025) (Table 2). In the current study group, 46 (64%) ACD titanium anchors were implanted in the *superior* EP with the polymer mesh linked to the *lower* EP of the adjacent vertebra. We analyzed median *upper* and *lower* EPC volumes in association to anchor localization (Fig. 4). A significant difference between the *inferior* and *superior* anchor localization was only present at the *lower* EP (p = 0.022).

**Table 1:** Results of the multivariate regression analysis of risk factors for EPC of the *lower* and *upper* endplate revealed only one significant parameter: anchor localization in *superior* endplate

Multivariate regression analysis	p-value
Sex (effect of male)	0.4502
Smoker	0.1487
Age	0.6961
Anchor localization *(superior)*	*0.0380*
Heavy lifting	0.2494
Amount of disc material removed	0.8110
Size of defect	0.5105
Preoperative disc height	0.6468
Postoperative Pfirrmann score	0.8367
Postoperative Modic score	0.7446

*EPC* endplate changes

**Table 2:** Univariate regression analysis and significance level of each predictor on *lower* EPC

Univariate regression analysis	p-value
Sex (effect of male)	0.9182
Smoker	0.4776
Age	0.4337
Anchor localization *(superior)*	*0.0252*
Heavy lifting	0.1245
Amount of disc material removed	0.6441
Size of defect	0.2446
Preoperative disc height	0.8333
Postoperative Pfirrmann score	0.5502
Postoperative Modic score	0.8817

### Descriptive score

This grading system was based on the fact that patients with ACD in *inferior* endplate had, on average, smaller EPC compared to *superior* EP fixation. Table 3 presents the descriptive criteria for the ACD—EPC scoring system. The scoring system was validated in our patient cohort and Table 4 presents the distribution in our study cohort.

**Table 3:** Introduction of a new classification of ACD associated EPC. The EPC score ranges from 1 to 4 and assesses anchor localization *(superior/inferior)* and size of EPC (< 1cm^3^ / > 1cm^3^) as criteria. Scores were defined as follows: grade 1 includes patients with ACD implanted on *inferior* EP and EPCs of < 1 cm^3^, grade 2 have an ACD in the *superior* EP and EPCs < 1 cm^3^, grade 3 received ACD in *inferior* EP and EPCs > 1 cm^3^, and finally maximum grade 4 if ACD is in the *superior* EP and the EPCs are > 1 cm^3^. ACD = Annular closure device

	Localization of the anchor	Size of the EPC
EPC classification		
Grade 1	Caudal	< 1 cm^3^
Grade 2	Cranial	< 1 cm^3^
Grade 3	Caudal	> 1 cm^3^
Grade 4	Cranial	> 1 cm^3^

**Table 4:** Validation of the EPC classification in our study cohort. A maximum achievable score of 4 points corresponds to severe EPC and the minimum of one point indicates mild EPC. Scores of 2 to 3 points are considered intermediate

I	II	III	IV
EPC grading in our cohort			
23	40	0	8

### Case report

**Fig. 5:**
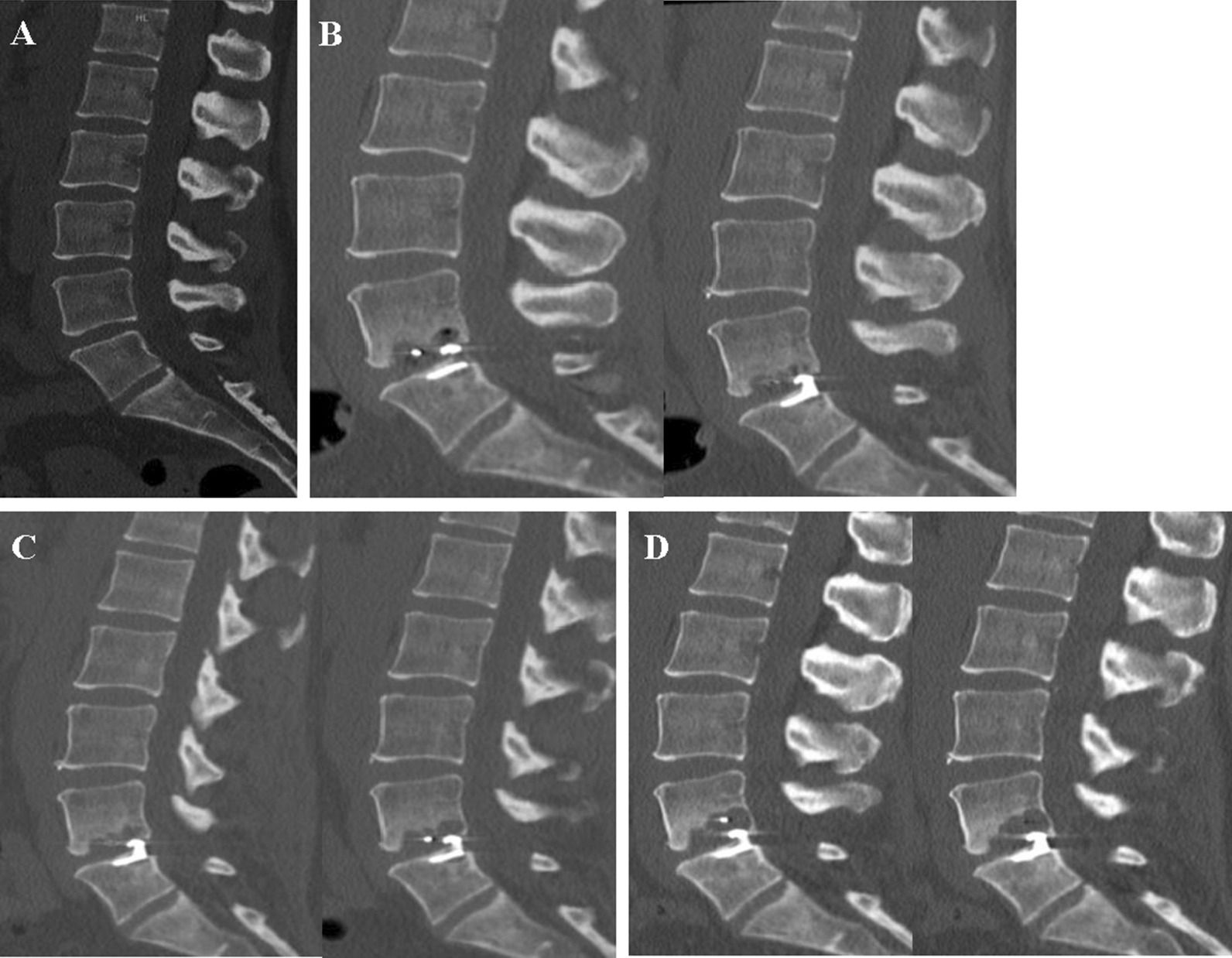
Case report of a patient that underwent LMD and ACD implantation at L4/5 level due to disc herniation. **a** Preoperative baseline CT: No EPC visible. **b** One-year follow-up: EPC (0.57 cm^3^) appear at the *lower* EP of L4. **c** 2-year follow-up: EPC (1.15 cm^3^) progressed at *lower* EP of L4. **d** 3-year follow-up: EPC (2.46 cm^3^) accelerated to a grade 3, with polymer mesh subsidence into *lower* EP of L4

Figure 5 illustrates a case of a 52 year-old women who underwent LMD and ACD implantation at L4/5 level due to disc herniation and persistent L5 radicular pain. Preoperative CT confirmed absence of any EPC. LMD and ACD implantation in the *superior* EP of L5 was successful, no complications occurred intraoperative and radicular pain was resolved postoperatively. At one-year follow-up, EPC (size = 0.57 cm^3^) had appeared around the mesh at the *lower* EP of L4. Only minimal changes were noticeable below the titanium anchor at *upper* EP of L5. After 2 years, EPC (size = 1.15 cm^3^) progressed in the *lower* EP of L4 and at the 3 years follow-up, EPC (2.46 cm^3^) increased to a grade 3, with polymer mesh subsidence into the *lower* EP of L4. Instability caused increasing facet joint-associated low back pain and the patient underwent subsequent fusion and removal of the implant.

### Reoperation due to reherniation or device failure

**Fig. 6:**
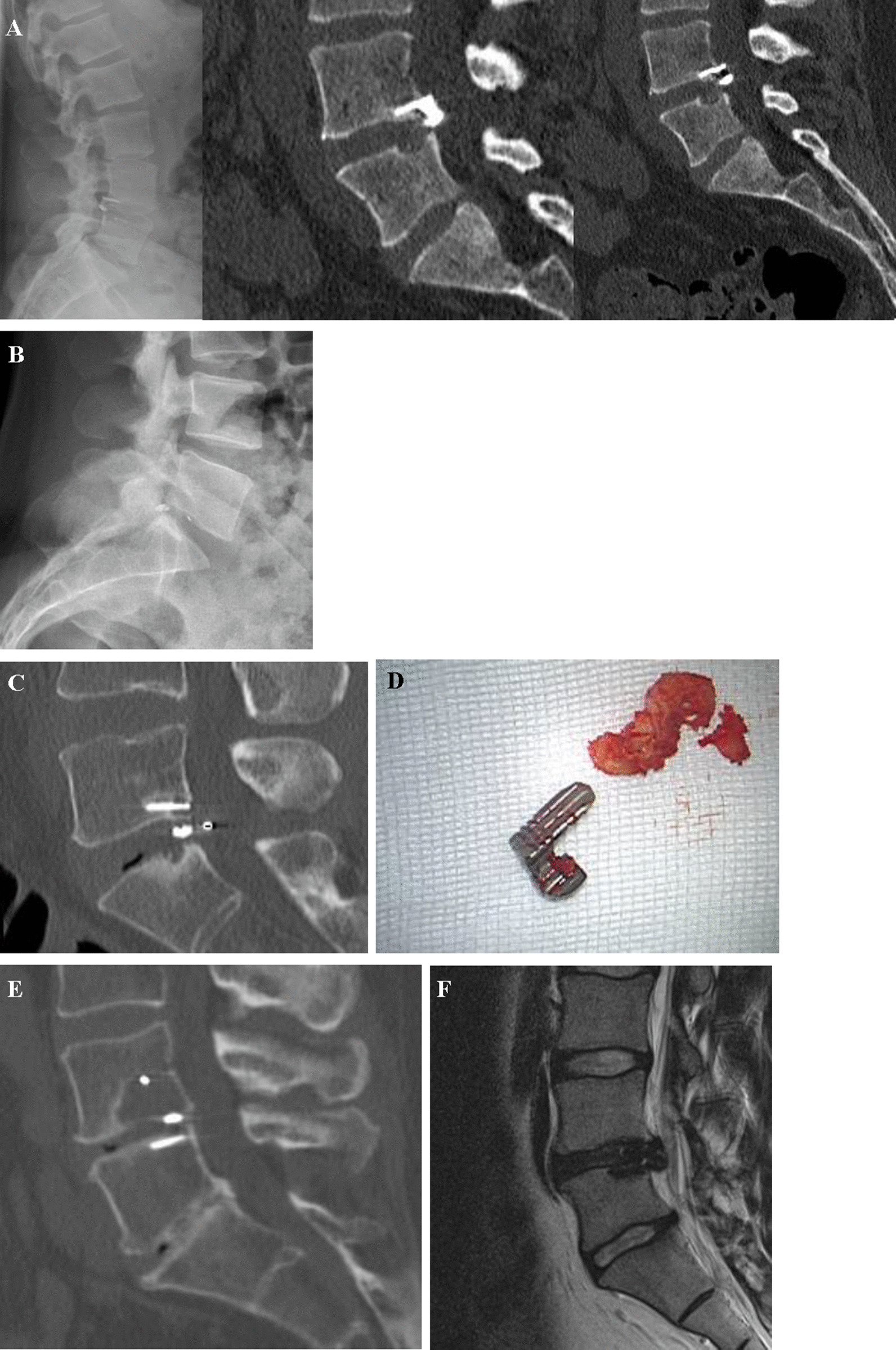
Several types of device failure are shown. **a** Dislocation of the whole device posterior into spinal canal. Patient underwent rediscectomy and ACD removal. **b** Device material failure: fracture of the titanium anchor-head and dislocation toward spinal canal. Rediscectomy and anchor head and mesh removal was performed. **c** Posterior dislocation of polymer mesh into spinal canal. A LMD and mesh removal was performed during revision surgery. **d** Intraoperative picture of removed ACD device. **e** Radiological appearance of mesh subsidence into EPC and vertebra osteolysis. Clinically asymptomatic finding, no reoperation necessary to-date. **f** ACD failure: symptomatic recurrent disc herniation at the index level leading to a reoperation with LMD only

At the time of study analysis in 2018, 65 patients (90%) were still living with the implant and in 7 (10%) patients the whole implant (n = 4), or only the polymer mesh (n = 3) had been removed. A total of 17 (24%) patients revealed reherniation in the postoperative follow-up MRI, 10 patients (13.8%) were symptomatic and 7 (10%) asymptomatic. In addition to conservative treatments (n = 4), reoperation was necessary in 6 (8.3%) patients in the symptomatic cohort. The procedures included recurrent LMD only (n = 3), LMD with ACD removal (n = 1) and LMD with mesh removal (n = 2). A device failure occurred in 19 (26.4%) patients. "Failure" was defined as: dislocation of the whole device > 2 mm (n = 5, 6.9%), device anchor-head breakage (n = 1, 1.3%), or posterior dislocation of the mesh into spinal canal (n = 13, 18%). Mesh subsidence into EPC was documented in 15 (20.8%) patients (Table 5 and Fig. 6). Overall, 7 patients (9.7%) underwent reoperation due to device failure within the follow-up period. In terms of reoperation techniques, the following surgical approaches were used in a total of 13 reoperations (18.1%): Re-LMD only (n = 3, 4%), removal of polymer mesh and LMD (n = 2, 3%), LMD with mesh and anchor-head removal (n = 1, 1%), explant of the whole ACD and LMD (n = 1, 1%), transpedicular fusion with ACD in place (n = 3, 4%) and fusion with ACD removal (n = 3, 4%) (Table 6). All reoperations occurred on average 18 ± 11 months after LMD and ACD implantation. According to the surgeons, reoperation and ACD removal was more difficult than re-LMD only. An unintended durotomy during reoperation occurred in 31% of cases. During all revision surgeries, portions of the mesh, bone and disc material were sent for bacteriological growth testing, and any ongoing infection was excluded.

**Table 5:** Types of surgical and device failure

Type of surgical and device failure	n (%)
Dislocation of whole ACD	5 (6.9)
ACD anchor head breakage	1 (1.3)
Posterior dislocation of mesh into spinal canal	13 (18)
Mesh subsidence into endplate	15 (20.8)
Symptomatic reherniation	10 (13.8)
Reoperation due to recurrent herniation	6 (8.3)
Reoperation due to device failure	7 (9.7)

**Table 6:** The patient subgroup that underwent reoperation due to recurrent disc herniation or device failure. Details of reoperation, implant approach and any complications are presented

Patient number	Age	Diagnosis	Type of recurrent surgery	ACD procedure	Time to reoperation (months)	Complication
Reoperations						
1	48	Recurrent herniation & mesh dislocation SC	LMD	Mesh removal	18	None
2	48	Instability	Fusion	None	24	None
3	30	Anchor head breakage	LMD	Mesh & anchor head removal	29	None
4	33	ACD dislocation	LMD	ACD removal	17	Dural injury
5	53	Instability	Fusion	None	46	None
6	57	Instability	Fusion	None	10	None
7	47	Recurrent herniation & mesh dislocation SC	Fusion	ACD removal	25	None
8	34	Recurrent herniation	LMD	None	15	None
9	40	Recurrent herniation	LMD	None	8	None
10	33	Recurrent herniation	LMD	None	14	Dural injury
11	32	Instability	Fusion	ACD removal	7	CSF leak, revision
12	51	Instability	Fusion	ACD removal	3	Dural injury
13	31	Recurrent herniation & mesh dislocation SC	LMD	Mesh removal	20	None

Of note: unintended durotomy occurred in 31% of reoperations. *SC* spinal canal

### Clinical outcome

**Fig. 7:**
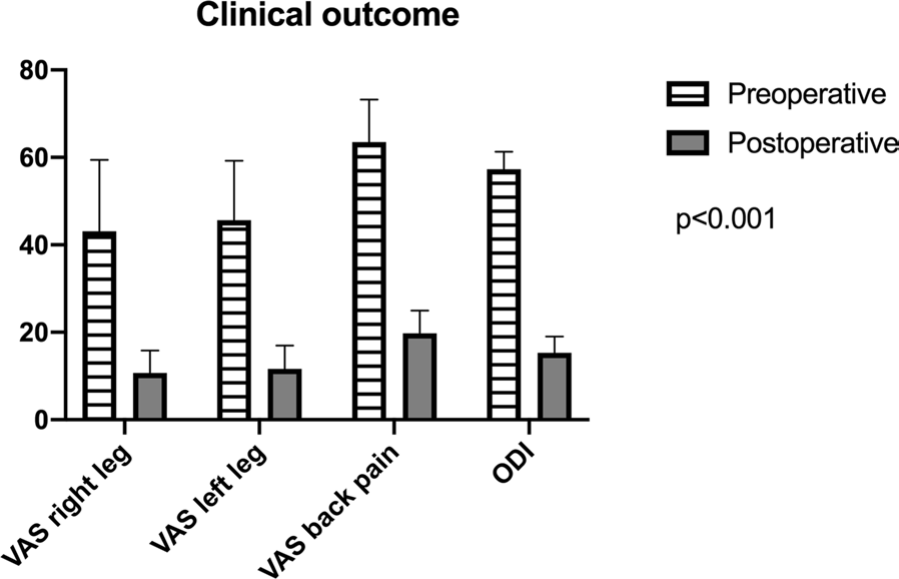
Clinical outcome **a** + **b** Regarding right and left leg pain VAS, a significant postoperative improvement could be shown. **c** Mean low back pain VAS improved significantly after surgery. **d** Significant improvement of mean postoperative ODI

In summary, the surgery achieved significant improvements with good clinical outcome. These findings are most likely independent from the ACD implantation and a normal consequence of LMD surgery. Mean preoperative ODI of 57.27 decreased significantly postoperative to a mean of 17.58 (95% CI [35.71, 46.99] p < 0.001). With regard to low back pain VAS, a preoperative mean of 63.53 showed significant postoperative reduction to 19.80 (95% CI [35.32, 57.26] p < 0.001). Left leg pain VAS, decreased significant from a mean of 45.66 preoperative to 11.46 postoperative (95% CI [23.72, 49.26] p < 0.001). Similarly, right leg pain VAS reduced from a preoperative mean of 42.97 to postoperative 10.79 (95% CI [21.45, 49.72] p < 0.001) (Fig. 7). We also investigated if the magnitude of EPC had any influence on postoperative outcome. In particular, *lower* EPC showed significant correlation with postoperative ODI at the last follow-up only (p = 0.01) and a trend was present with *lower* EPC and last postoperative low back pain VAS (p = 0.6). In fact, EPC do not seem to affect postoperative clinical outcome in a significant manner.

### Disc degeneration

**Fig. 8:**
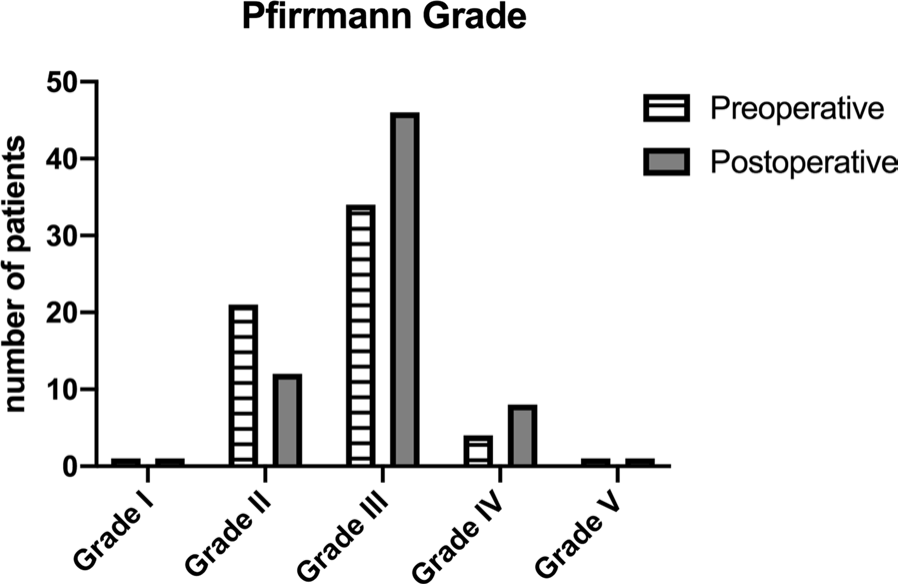
Assignment of our study cohort according to Pfirrmann classification. The majority intervertebral discs were classified as grade III before and after surgery

**Fig. 9:**
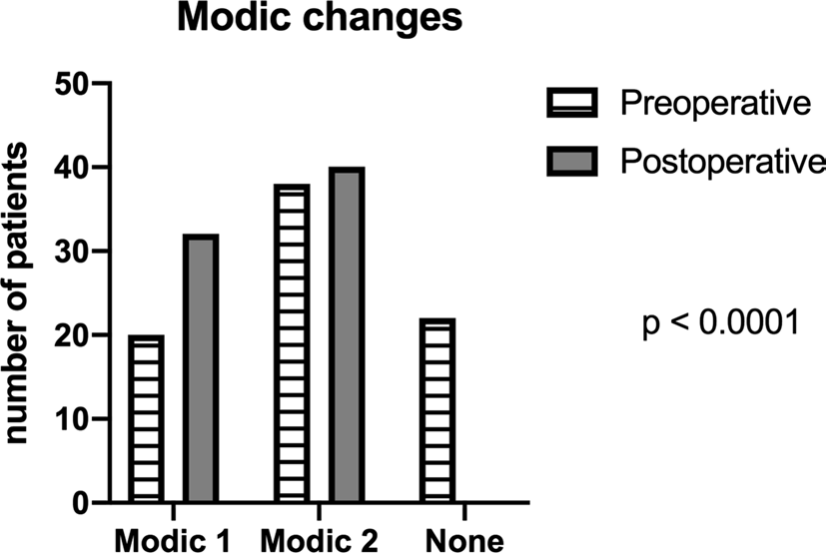
Assessment of endplate changes according to Modic classification in the current study patients. The amount of patients having Modic changes and specifically Type 1 Modic changes, increased after surgery. Number of Type 2 Modic changes was stable, and no Type 3 Modic changes were seen

MRI findings prior and after surgery were compared with a mean postoperative follow-up of 22.7 ± 9.7 months to assess DDD. Preoperative mean disc height measured 6.2 ± 1 mm and decreased postoperatively to a mean of 5.5 mm ± 1 mm. This 0.7 ± 0.6 mm reduction in disc height was non-significant. Disc degeneration at index level was Pfirrmann grade III in 49% preoperative and 66% postoperative (Fig. 8). A third of patients (31%) had no Modic changes before surgery, and Type 1 increased from 14 to 44% (Fig. 9).

## Discussion

The findings of this study support nascent evidence that ACD implantation after LMD might accelerate EPC and lead to osteolysis of the surrounding bone. In this study, EPC was documented in almost all patients during follow-up. Nevertheless, no impact on clinical outcome was apparent.

The present study revealed a reherniation rate of 14%, which is in the 3%-18% range following LMD reported in the literature [2]. The 3 year follow-up results from the multicenter randomized controlled European trial (RCT LMD—ACD), comparing LMD and ACD versus LMD only showed a symptomatic reherniation rate of 14.8% in the ACD group, versus 29.5% in the control group [12].

Our reoperation rate of 18% during the 18-months postoperative period was above reported average between 1 and 11.5% [1, 26]. In comparison, the RCT LMD—ACD trial showed that index level reoperations were significantly less frequent with ACD (9% vs. 16% after 2 years and 11% vs. 19.3% after 3 years) [12, 23].

Other studies have also reported the appearance of EPC after LMD and ACD [14]. One case report described ACD removal and fusion 1.5 months after surgery due to resorbed bone in vicinity of the device and inflammatory changes in the adjacent tissue [13]. In the RCT LMD – ACD study, EPC were significantly more prevalent in the ACD group compared to the control group (84% vs. 30% after 2 years and 89% vs. 41% after 3 years) [12, 23]. At baseline, the number of patients showing EPC was similar in both groups [5]. The number and total EPC volume increased more significantly in the ACD group over time [5]. Barth et al. had reported earlier that ACD implantation in the *superior* EP causes more frequent and larger EPC in the opposite *lower* EP due to applied pressure from the mesh [5]. Indeed, the *lower* endplate seems to be more affected by DDD and ongoing tissue damage.[3].

Endplate changes themselves are a common finding after LMD only, with a reported incidence of 43%—57% [4, 25]. There might be an association in LMD between surgical technique and aggressive treatment of sensitive EP. Earlier studies have shown, that endplate changes and DDD are related [8, 21]. In the present study, no relevant increase of DDD grade was seen during follow up.

In cases with ACD, the flexible polymer mesh might cause friction, leading to EPC around the mesh. More research and improvement of implant design is needed. Although anatomy mainly determines the anchor position, in general, ACD implantation in the *superior* EP should be avoided.

Lange et al. recently reported on a symptomatic ACD loosening due to infection with Propionibacterium acnes, which they confirmed after revision surgery [15]. In our series, disc material cultures and device sonification ruled out any low-grade infection as a possible cause of EPC.

A descriptive classification was introduced to facilitate interprofessional communication about ACD-associated EPC. This scoring system was validated in our patient cohort but further external score validation will be necessary.

Finally, the risk of EPC must be balanced against a possible lower reherniation rate. Based on our experience, we would suggest that only patients with a large annular defect (> 6 mm) and high remaining disc volume should be considered for ACD implantation on the *inferior* EP. Of higher importance is an improvement of surgical techniques to achieve smaller annular incisions (< 6 mm) in order to prevent reherniation.

### Study limitations

Limitations of our current study are the fact that this is a non-comparative single center study with a small patient cohort.

## Conclusion

There was significant postoperative clinical improvement after limited LMD and ACD implantation. The high incidence and volume of EPC did not affect clinical outcome. The ACD might prevent disc reherniation despite implant failure rates. Long-term clinical and radiological assessments is necessary to evaluate the consequences of these findings.

## Data Availability

This study includes data of a post-marketing, prospective, multicenter, randomized controlled trial (RCT) of limited discectomy—with and without use of an annular closure device (Clinicaltrials.gov NCT01283438) undergoing surgical treatment at our Institution. All data generated or analyzed during this study are included in this published article and can be made available from the corresponding author on reasonable request.

## Abbreviations

ACD: Annular Closure Device
CT: Computed Tomography
EPC: Endplate Changes
EP: Endplate
DDD: Degenerative Disc Disease
LMD: Limited Microdiscectomy
MRI: Magnetic Resonance Imaging
ODI: Oswestry Disability Index
RCT: Randomized Controlled Trial
VAS: Visual Analogue Scale
